# Real-World Walking Speed Assessment Using a Mass-Market RTK-GNSS Receiver

**DOI:** 10.3389/fbioe.2022.873202

**Published:** 2022-03-30

**Authors:** Luca Reggi, Luca Palmerini, Lorenzo Chiari, Sabato Mellone

**Affiliations:** ^1^ Health Sciences and Technologies-Interdepartmental Center for Industrial Research (CIRI-SDV), University of Bologna, Bologna, Italy; ^2^ Department of Electrical, Electronic, and Information Engineering “Guglielmo Marconi”, University of Bologna, Bologna, Italy

**Keywords:** real-world walking speed, remote monitoring, global navigation satellite system, real-time kinematic, validation

## Abstract

Walking speed is an important clinical parameter because it sums up the ability to move and predicts adverse outcomes. However, usually measured inside the clinics, it can suffer from poor ecological validity. Wearable devices such as global positioning systems (GPS) can be used to measure real-world walking speed. Still, the accuracy of GPS systems decreases in environments with poor sky visibility. This work tests a solution based on a mass-market, real-time kinematic receiver (RTK), overcoming such limitations. Seven participants walked a predefined path composed of tracts with different sky visibility. The walking speed was calculated by the RTK and compared with a reference value calculated using an odometer and a stopwatch. Despite tracts with totally obstructed visibility, the correlation between the receiver and the reference system was high (0.82 considering all tracts and 0.93 considering high-quality tracts). Similarly, a Bland Altman analysis showed a minimal detectable change of 0.12 m/s in the general case and 0.07 m/s considering only high-quality tracts. This work demonstrates the feasibility and validity of the presented device for the measurement of real-world walking speed, even in tracts with high interference. These findings pave the way for clinical use of the proposed device to measure walking speed in the real world, thus enabling digital remote monitoring of locomotor function. Several populations may benefit from similar devices, including older people at a high risk of fall, people with neurological diseases, and people following a rehabilitation intervention.

## Introduction

The ability to move is a feature that characterizes most of the animal kingdom because it plays a critical role in finding food, escaping danger, and surviving. Notably, generalized slowing of movement is associated with aging (Studenski, 2009). Even for humans, where these primordial tasks are less important, the capacity to move is essential to maintain independence in daily activities (Yildiz, 2012) and good quality of life (King et al., 2013). From a physiological point of view, locomotion is a complex matter involving the following systems: nervous (central and peripheral), perceptual, muscular, and skeletal. It is also influenced by how energy is produced and delivered (Ferrucci et al., 2000). One of the most significant parameters summarizing the ability to move is walking speed (WS) (Abellan Van Kan et al., 2009), a valid, sensitive, and specific measure (Fritz and Lusardi, 2009). It is widely used as a predictive tool for future adverse outcomes such as disability (Newman et al., 2006), shortened survival time (Studenski, 2011), institutionalization (Montero-Odasso et al., 2005), worsening health status (Studenski et al., 2003), and falls (Guimaraes and Isaacs, 1980). Walking speed reflects both functional and physiological changes (Perry et al., 1995).

For this reason, it is useful to evaluate the effects of rehabilitation (Goldie et al., 1996). In fact, several medical fields such as neurology, geriatrics, orthopedics, and cardiology assess WS (Graham et al., 2008). A WS measurement can be part of a more structured and validated performance test (Schimpl et al., 2011) (e.g., Short Physical Performance Battery and Time Up and Go), or be used as a single measure, especially for predictive purposes (Hardy et al., 2007).

The WS is primarily assessed over short distances (both for ease and speed of execution), typically four or 10 m (Graham et al., 2008); a stopwatch is used to measure the time taken to travel the assigned distance. The measure is performed predominantly in a controlled environment. While this setup is prevalent and validated, some studies have pointed out the poor ecological validity of assessing WS in an environment like a hospital or clinical facility (Moseley et al., 2004; Stellmann et al., 2015). In fact, unlike walking in real life, the distance is limited, and the environmental conditions are relatively unvarying. As a result, different technologies, like inertial measurement units (IMU) or global positioning systems (GPS), have been used to measure walking speed continuously in daily life. The latter solution offers distinct advantages, as it allows to have a direct and continuous measurement of WS without the need for integration (e.g., accelerometer) (Gernigon et al., 2015) or supervision (Gernigon et al., 2014). Besides WS, it allows, using the absolute position, an assessment of different types of mobility patterns (Fillekes et al., 2019) (e.g., for epidemiological studies (Klous et al., 2017)). Previously, Le Faucheur et al. (2007), Townshen et al. (2008), and Noury-Desvaux et al. (2011), tested the accuracy of portable, low-cost stand-alone GPS devices in measuring walking speed in environments with complete sky visibility (e.g., outdoor running track, public park free of buildings and dense trees), obtaining promising accuracy. One of the major limitations of such devices, however, is that their accuracy decreases in environments where obstacles obstruct sky visibility (like under trees or near high buildings) and becomes very low when used indoors. Still, those environments with decreased visibility are also very important for a real-world evaluation of walking. One technique that increases the system’s accuracy, especially in the situation of decreased sky visibility, is the Real Time Kinematic (RTK) modality, based on the use of two communicating GPS receivers to obtain a differential solution (which standard stand-alone GPS devices cannot perform). This technique is not new, but until a few years ago only bulky, very expensive (>1000 €) survey-grade receivers were available. Recently, the mass-market production of RTK-capable chipsets has reduced costs and dimensions while ensuring good accuracy (although lower than professional receivers).

This work aims to assess the suitability of a mass-market RTK receiver to measure real-world walking speed (RWWS), in a challenging environment with different degrees of sky visibility. To do so, the data from the RTK receiver is processed as detailed below and compared with values obtained from typical reference systems for these kind of studies (an odometer and a stopwatch).

## Materials and Methods

### Instrumentation and Data

The tested device is a low-cost (∼250€), dual-band, multi-constellation RTK-capable receiver called simpleRTK2B (Ardusimple, Spain), based on the chipset ZED-F9P (u-blox). This device was the first mass-market receiver with upper and lower L-band coverage for all major constellations of GPS, GLONASS, Galileo, and BeiDou satellites, collectively known as GNSS (Global Navigation Satellite System). The RTK technology is based on the communication between two receivers, a base, and a rover. The base receiver, located in a fixed, known position, can calculate the error between its actual position and its position as estimated by the GNSS. If the base and rover are close enough (less than 30 km), they suffer the same environmental errors, so the rover can use the error calculated by the base as a differential correction to improve its accuracy.

The receivers communicate through the internet as caster and client, using the NTRIP protocol (Dammalage and Samarakoon, 2008). In our study, the simpleRTK2B worn by the participants acted as the rover. We used a reference station (part of the EUREF permanent GNSS network) that offers a no-fee caster service as the fixed base. The base station, located in Medicina (BO, ITA), uses a LEICA GR25 receiver situated about 25 km away from the testing location (Costa-Saragozza district, BO, ITA). We used the Lefebure NTRIP client application, installed on a smartphone (Oneplus 6) and connected to the receiver through Bluetooth, to receive the differential corrections and log the NMEA sentences obtained from the receiver (u-bloxF9HPG, 2021).

**FIGURE 1:**
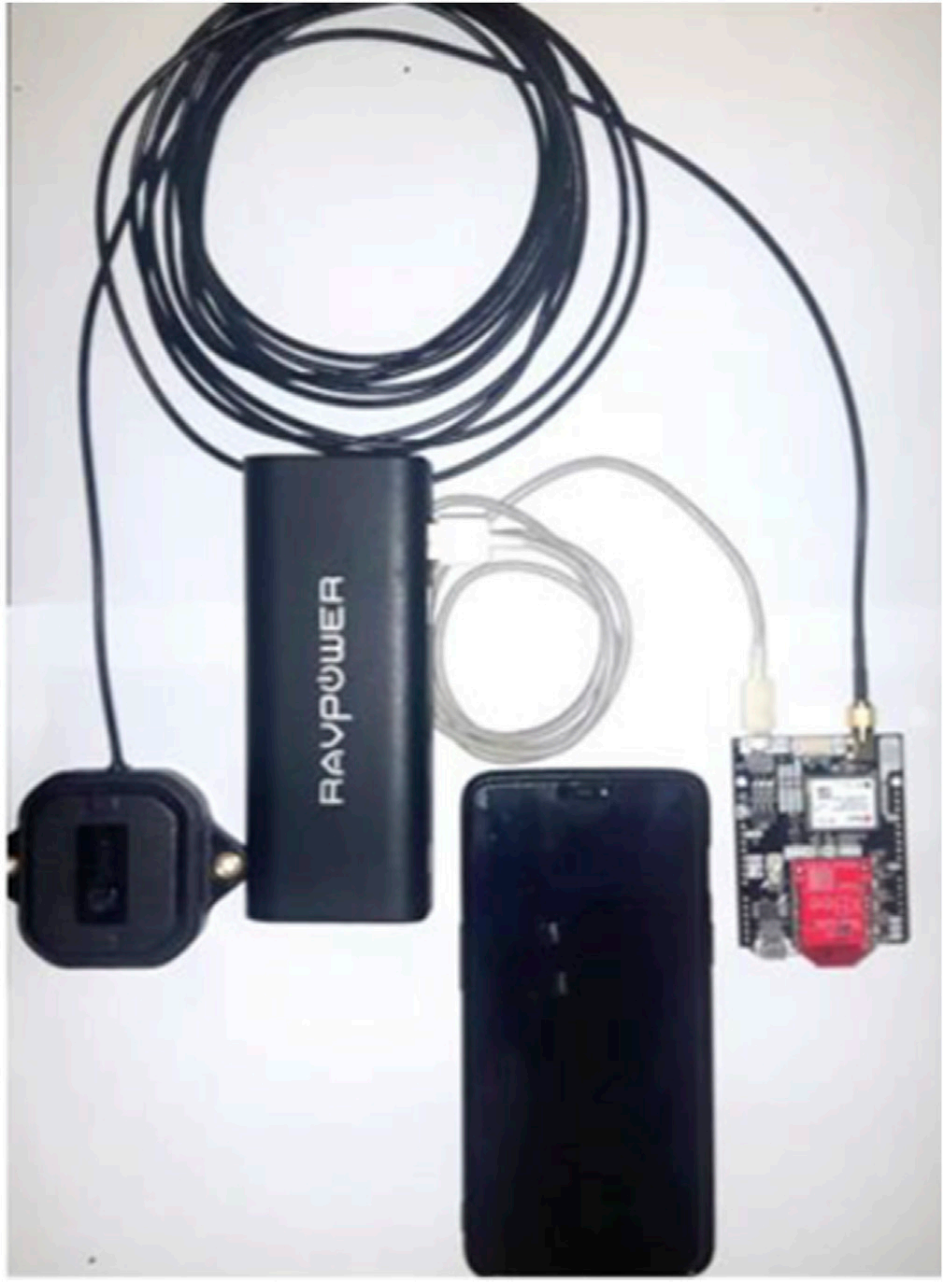
Device set-up: simpleRTK2B, antenna, power bank, and smartphone.

The rover setup comprised the simpleRTK2B (weight: 100 g; dimension: 68 × 53 mm) wired with an antenna (ANN-MB series, u-blox, weight: 200g; dimension: 60 × 55 mm) and powered by a power bank (see Figure 1).

We were interested in extracting only two NMEA sentences from the RTK rover: the RMC (Recommended Minimum Specific GNNS Data) and GGA (Global Positioning System Fixed Data). For this reason, we set the device to output only these sentences, at the minimum sampling period allowed, 55 ms (18.18 Hz). From these sentences, we extracted the data shown in Table 1 for each sample.

**TABLE 1:** Data extracted from NMEA sentences.

Description	Source	Format	Units
Latitude/longitude	RMC	ddmm.mmmm/dddmm.mmmm	Degrees and decimal minutes
UTC Time	RMC	hhmmss.sss	Hour, minute, second, millisecond
Speed over ground	RMC	—	knots
Status: presence of the solutions	RMC	‘V’ for void or ‘A’ active	—
MLS altitude	GGA	—	m
Age of differential corrections: seconds since the last update of the corrections by the References station	GGA	—	s
Horizontal dilution of precision (HDOP): effect of navigation satellite geometry on positional measurement precision	GGA	—	—
Satellites used: number of satellites used in the solution (for the NMEA format, the maximum number is 12)	GGA	—	—
Position fix indicator: modality in which the device calculated the solution (RTK, float RTK, DGPS, or GPS: defined below)	GGA	—	—

The number of satellites used, the age of the differential corrections and the HDOP (Horizontal Dilution Of Precision) were recorded to monitor the receiver’s functioning during the acquisitions. However, since their values were almost always constant and good, they will not be presented in the following analysis. Also, the altitude is not mentioned further because the test took place in a flat area.

The device can work in different modalities (called position fixed indicator) with different qualities (and corresponding degrees of accuracy). This is because calculations are performed differently for different modalities. Each modality is summarized here in descending order of accuracy (quality):

1. RTK: differential technique based on carrier signal with an integer resolution of the integer ambiguity (Teunissen, 2003).
2. Float RTK: differential technique based on carrier signal with a float resolution of the integer ambiguity.
3. DGPS: differential technique based on code signal.
4. GPS: single-point positioning, stand-alone functioning. For a more detailed explanation, please refer to (Kaplan and Hegarty, 2005).

### Experimental Protocol

**FIGURE 2:**
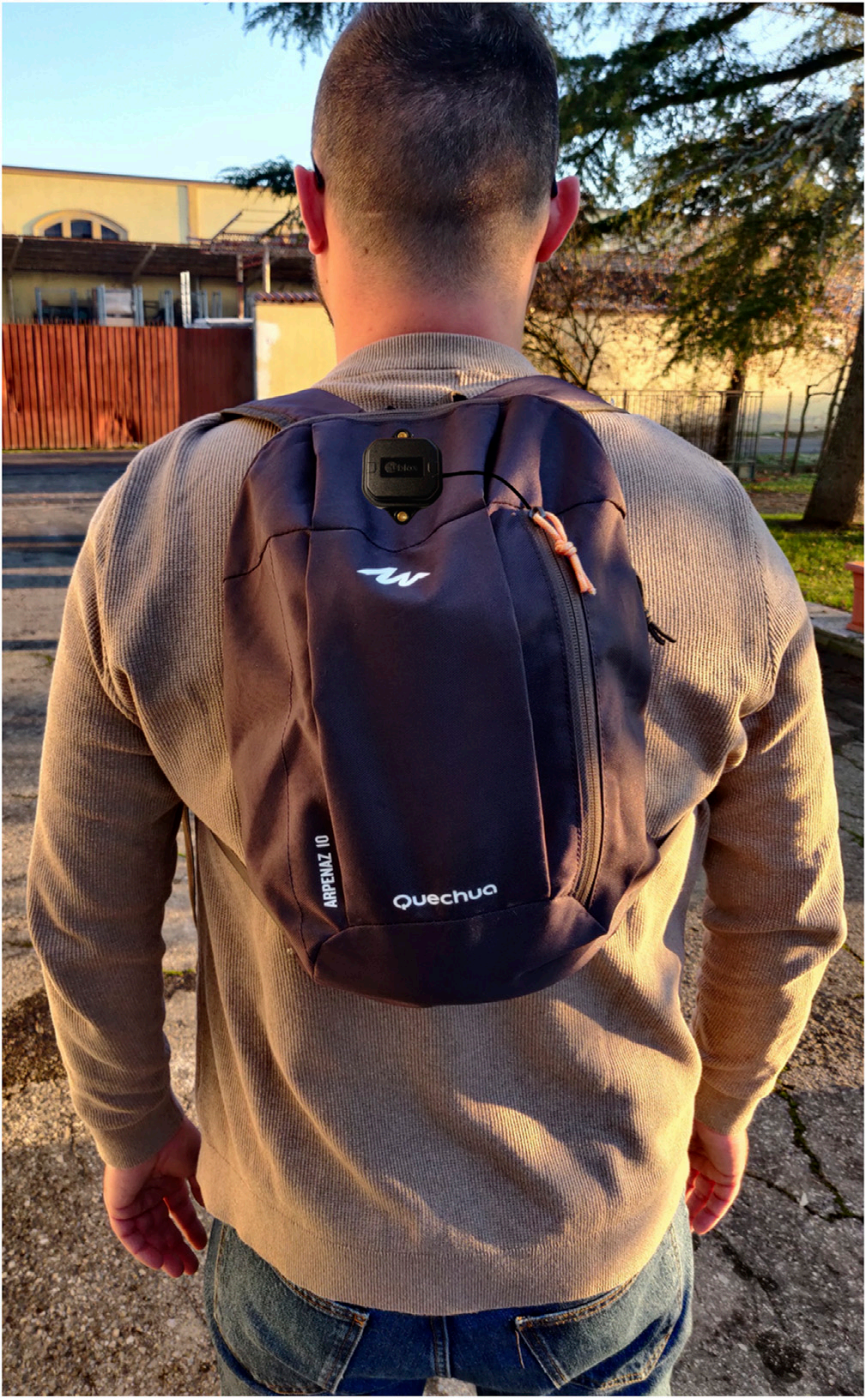
Set-up of a participant.

**FIGURE 3:**
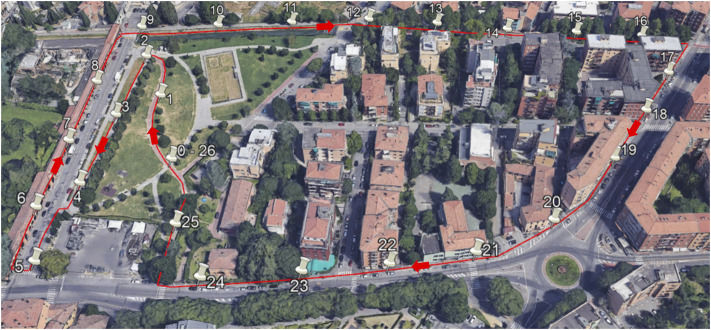
Image taken from Google Earth showing the 1300-m path (red line) and the start and end points of each walking tract (white pins). Description of the tracts: 1–5 park (open sky), 6–9 arcades (totally obstructed visibility), 10–12 bicycle lane (open sky with a few trees), 13–17 bicycle lane under tall trees, with tall buildings on both side (highly obstructed visibility), 18–24 sidewalk with tall buildings (partially obstructed visibility), and 25–26 park (open sky with a few trees).

Seven healthy adults, five males and two females (age: 32 ± 6 years, height: 174 ± 14 cm), were recruited for this study after giving their informed consent. The acquisitions were all made on the same day (from 9:30 a.m. to 2 p.m.), with sunny and cloudless environmental conditions. Participants had the device in a backpack with the antenna on it (see Figure 2), similarly to previous studies (Le Faucheur et al., 2007; Noury-Desvaux et al., 2011). To test the accuracy of the device in different operating conditions, we defined a flat, 1300-m loop with different sky views (see Figure 3): open sky (park); open sky with few interferences (few trees), highly obstructed (under tall trees with buildings on both sides), and totally obstructed (under arcades). Figure 3 shows a photo of the path with pins describing the start and endpoints of the 26 tracts (each tract is described by two pins, e.g., tract 1 is between pin 0 and 1). This area is in the Costa-Saragozza district of Bologna (lat: 44.495,280°, long: 11.312,900°). Participants started walking in an open field to allow the device to operate with the highest accuracy possible and then walked 26 tracts of approximately 50 m (49.9 ± 1.7 m) at a comfortable pace while carrying the backpack and an odometer (STANLEY MW40M, accuracy 1 dm). This instrument accurately measured the distance and showed the participants and the examiner when they had walked 50 m and had to stop. An examiner walking beside the participant timed each tract with a stopwatch (Finis 3 × 100M, accuracy 1/100s). At the end of each tract, when the examiner instructed the participant to stop, the examiner noted down the elapsed time, and the distance traveled.

### Data Processing

**FIGURE 4:**
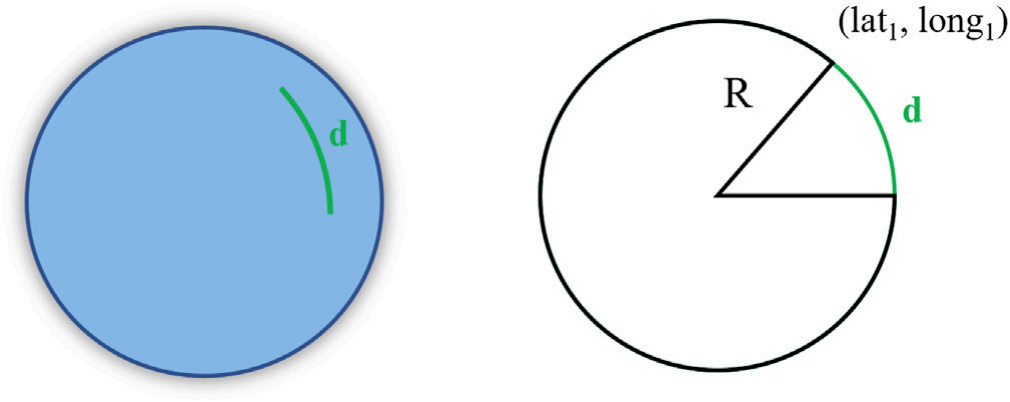
Graphical explanation of the haversine formula.

First, we discarded the sentences with ‘V’ in the RMC status field, indicating the receiver did not obtain a result. This can happen for many reasons, like very poor coverage. We converted latitude, longitude, time, and velocity from the NMEA formats to degree, DateTime format, and m/s, respectively. By manually inspecting the raw velocity signal, we found the start and end of each 50-m tract and extracted the walking sessions from the resting periods for a total of 182 (26 × 7) sessions whose average speeds could be compared. We calculated the elapsed time, and the distance traveled for each walking session. The distance was calculated as the line integral between the start and end of the walking session. More precisely, we summed all the distances (D) calculated between each position sample (lat, long) using the haversine formula:

$$D=2*R*\arcsin\left(\sqrt{\sin^{2}\left(\frac{lat_{1}-lat_{2}}{2}\right)+\cos\left(lat_{1}\right)*\cos\left(lat_{2}\right)*\sin^{2}\left(\frac{long_{1}-long_{2}}{2}\right)}\right)$$ (1)

where *R* is the radius of the earth (6,371 km), *lat* is the latitude and *long* is the longitude of each of the two points between which we calculate the distance (see Figure 4).

We calculated the average RWWS measured by the simpleRTK2B as the distance walked divided by the elapsed time. We chose this method to be consistent with the odometer reference system, which uses the same formula. In fact, for the reference values, the distances obtained from the odometer were divided by the times obtained from the stopwatch.

### Statistical Analysis

The comparison between the average RWWS measured with the simpleRTK2B and the reference system was performed using the Pearson’s correlation coefficient to measure the linear association between the two sets of data, the R-squared value to quantify the explained variance, and the Bland-Altman analysis (Bland and Altman, 1999) to test the agreement between the two systems. The minimal detectable change (MDC) was also calculated to measure the system’s accuracy. It was calculated as one-half the difference between the Bland-Altman plot’s upper and lower agreement limits (Haghayegh et al., 2020). Finally, to better understand the relationship between the accuracy of the measurement and the different working modalities, which are influenced by the degree of visibility of the sky in the different tracts, we repeated the same analyses on two subsets of the data. These cases were created by discarding all the values obtained in tracts with the percentage of DGPS solutions (the least accurate modality achieved during the study) above 50 and 25%, respectively.

A bootstrap analysis (using random sampling with replacement repeated 100 times) was performed to evaluate the confidence intervals of the obtained metrics. It was also used, together with one-way analysis of variance (ANOVA) and Tukey-Kramer test for multiple comparisons, to evaluate significant differences between the three cases (the full dataset and the two subsets with different quality thresholds).

The analyses were performed in Matlab (R2020a).

## Results

**FIGURE 5:**
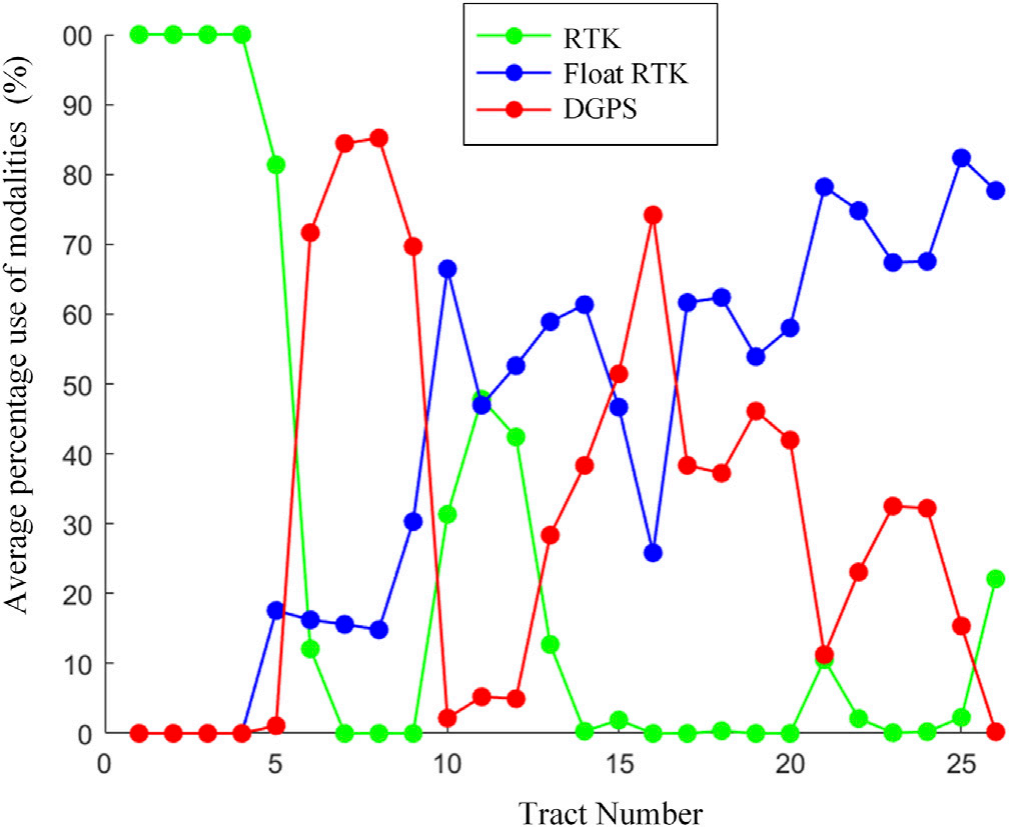
Average percentage use of the different modalities in each track.

During the experiment, the device worked in three modalities (RTK, float RTK, and DGPS) for 23.8%, 43.4%, and 32.8% of the time, respectively. It never worked in the GPS modality. The behavior of the simpleRTK2B receiver in the different tracts of the path is described in Figure 5, which shows the average percentage usage of the three modalities achieved for each tract. The RTK modality (highest quality) is the most difficult to obtain, and, as expected, it is mostly achieved in tracts with open sky visibility (for example, tracts 1–5 and 10–12). On the other hand, as expected, the DGPS modality is mainly present in tracts with total obstructed sky visibility like arcades (tracts 6–9) or poor visibility due to tall trees and/or buildings (tracts 14–20). Instead, the float RTK modality is often present across all tracts, especially in tracts with a partially obstructed sky view.

Another aspect worth mentioning about the device behavior is the low number of empty results from the device (discarded in pre-processing). Out of 82,171 walking samples, only 95 (0.1%) were discarded, and 64 of them belonged to a single participant (Participant 1). As expected, 93% of these data points happened while the participants were under the arcades.

**FIGURE 6:**
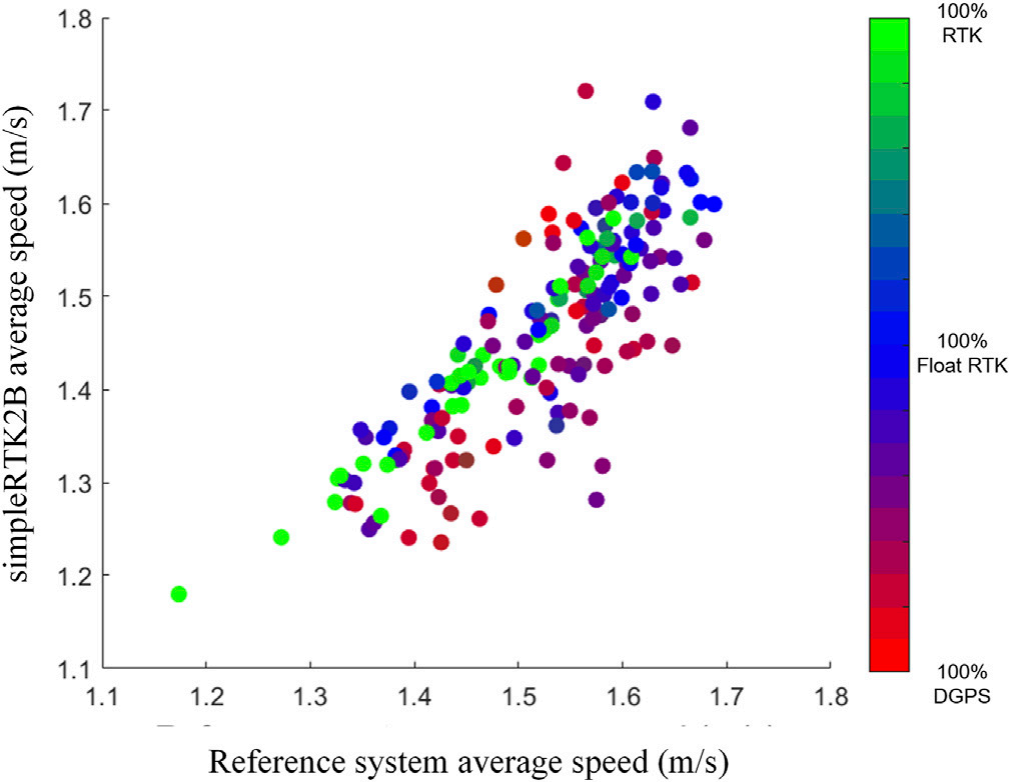
Scatter plot of the average walking speed (*x*-axis reference, *y*-axis GPS). The color map represents the RGB triplets associated with the relative frequency of the three working modalities in each tract (RGB) = (n° DGPS, n° RTK, n° float RTK).

The two obtained walking speed datasets (simpleRTK2B and reference system), and the related scatter plot are presented in Table 2 and Figure 6. In Figure 6, color represents the average quality of the tract in which that speed calculation was performed.

**TABLE 2:** Walking speed values obtained from the RTK and the reference systems.

Participant	simpleRTK2B Speed, m/s (std) [Range]	Reference Speed, m/s (std) [Range]
1	1.55 (0.07) [1.44–1.72]	1.59 (0.06) [1.47–1.68]
2	1.50 (0.07) [1.37–1.64]	1.55 (0.05) [1.46–1.69]
3	1.32 (0.06) [1.18–1.48]	1.37 (0.06) [1.17–1.47]
4	1.40 (0.06) [1.27–1.49]	1.47 (0.04) [1.37–1.56]
5	1.48 (0.10) [1.24–1.65]	1.56 (0.08) [1.33–1.67]
6	1.43 (0.12) [1.24–1.71]	1.51 (0.10) [1.27–1.66]
7	1.52 (0.08) [1.32–1.63]	1.58 (0.05) [1.45–1.66]
Tot	1.46 (0.11) [1.18–1.72]	1.52 (0.10) [1.17–1.69]

The average walking speed, its standard deviation (std), and range [min–max] for both the simpleRTK2B and reference systems, considering all 26 measurements from each subject. In the last line (Tot), values are calculated considering all the tracts of all the participants.

The Pearson’s coefficient, the R-squared, and the MDC obtained from the Bland-Altman analysis are shown in Table 3, divided into the three cases considered (full dataset and the two subsets with different quality thresholds). For the two cases in which the quality threshold is applied, the number of discarded tracts, the average (±std) number of tracts discarded for each participant, and the remaining tracts in which WS is calculated are also reported.

**TABLE 3:** Summary results.

	Pearson’s Coefficient [CI]	R-squared [CI]	MDC (m/s) [CI]	Total Number of Discarded Tracts	Number of Tracts Discarded by Participant (mean ± std)	Number of Tracts where WS is Calculated
All values	0.82 [0.77 0.88] *	0.67 [ 0.59 0.76] *	0.12 [0.11 0.14] *	0	0	182
DGPS <50%	0.9 [0.84 0.95] *	0.81 [0.71 0.90] *	0.09 [0.07 0.11] *	52	7 ± 2	130
DGPS <25%	0.93 [0.91 0.96] *	0.87 [0.82 0.93] *	0.07 [0.06 0.09] *	90	13 ± 3	92

Summary of the computed metrics for the three cases: values from all tracts, values from tracts with <50% DGPS solutions, and values from tracts with <25% DGPS solutions. The MDC is calculated as one-half the difference between the upper and lower limits of agreement of the Bland-Altman plot. CI: 95% confidence intervals obtained with bootstrap sampling; * significant difference (*p* < 0.001) from both the other groups.

**FIGURE 7:**
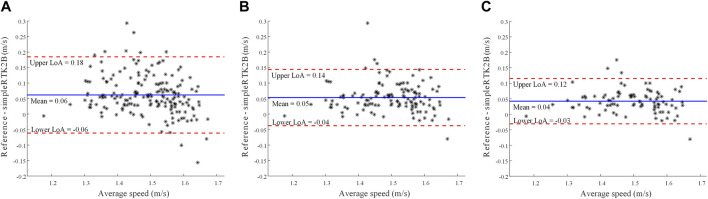
Bland-Altman plots considering all tracts **(A)**, tracts with <50% DGPS solutions **(B)**, and tracts with <25% DGPS solutions **(C)**. The mean difference and the upper and lower limits of agreements (LoA) are reported.

Lastly, Figure 7 presents the Bland-Altman plots with the average difference values and the limits of agreement for the three cases.

## Discussion

Multiple reasons led the device to work in the different modalities presented above, such as the base station receiver type, coordinate accuracy, number of available satellites, environmental factors, and operating range.

Within the environmental factors, in this work we mainly considered landscape-related factors, such as trees and buildings, which may obstruct the signal and lead to multipath interference (reception of reflected signals). The path selected for the acquisition offered different-use scenarios typical of a city, like tall buildings, arcades, and trees which entirely or partially obstructed visibility (obstructions could also be underpasses or hallways between buildings). On the other hand, the path also presents tracts with open sky typical of a city park and situations of mild interference. Such a heterogeneous scenario is an excellent and challenging test for a GPS receiver.

First, it should be noted that although a tiny percentage of invalid points occurred in the tracts with high interference, the simpleRTK2B never selected the lowest-quality modality (GPS), which is a single-point positioning modality that would be equal to standard GPS stand-alone devices. Instead, the device always worked in a differential configuration. This result reflects the advantage of using an RTK receiver instead of a classic stand-alone GNSS logger or a smartphone for monitoring purposes since the differential modality leads to better accuracy than single-point positioning. Considering that among the differential modalities, the RTK is better than DGPS,, another positive aspect is that the device worked for 43.4% in float RTK and for 23.8% in RTK.

The comparison with the reference system shows a pretty good correlation (Pearson’s coefficient of 0.82), even though the GPS receiver tended to underestimate the RWWS, as we can see in Figures 6, 7 and Table 2. This tendency is higher for speed values obtained with lower accuracy (red and violet shades in Figure 5).

When applying the two quality thresholds, the correlation coefficient increases to 0.93. If we look at the scatter plot, we can see that the green and blue points tend to be closer to the equality line. Similarly, for the R-squared values, the explained variance goes from 0.67 to 0.87 using the quality thresholds. This statistically significant improving trend (correlation and R-squared values are significantly higher, as expected, with higher quality) demonstrates how the different sky visibilities influence the system’s functioning.

The MDC obtained was 0.12 m/s in the general case, with [0.11 0.14] as confidence interval. A recent review (Bohannon and Glenney, 2014) found minimal clinically important differences (MCIDs) for comfortable speed to be in a range between 0.1 and 0.17 m/s for various pathologies. Previous studies also indicated that an improvement or decrease of 0.1 m/s is related to positive or negative health outcomes, respectively (Purser et al., 2005; Hardy et al., 2007; Fritz and Lusardi, 2009). Therefore, the obtained MDC for the general case (and corresponding confidence interval) is in line with the reported MCID values, indicating a promising capability in identifying a clinically meaningful change in walking speed. Furthermore, the values, 0.9 m/s and 0.7 m/s, achieved from the two higher quality subsets of data perform even better, being under the reported MCID range, with the latter having the whole confidence interval below the range. The MDC is significantly lower (as expected) with higher quality.

One aspect to consider about these results is that the MCID are obtained from measurements conducted in the laboratory setting and not in the real-world environment, where values of MCID of walking speed are still not known. Further studies should consider how/whether the reported MCID values would change in the real world.

So, the procedure of applying a threshold on the quality of the tracts could be useful to increase the system’s accuracy in obtaining the average speed of the entire path. Still, it could be argued that by applying this threshold, some tracts are discarded from the computation, and this could be a problem if the presence of discarded tracts is significant. Further studies on this aspect may be needed.

The results of the agreement analysis are in line with the ones obtained by Le Faucheur et al. (Le Faucheur et al., 2007), who used a low-cost non-differential stand-alone GPS. Also, they tested the device on an outdoor running track, which has complete sky visibility, while we tested the presented device in an environment with different degrees of sky visibility and interference.

In our study, the Bland Altman plots underline a small (between 0.04 and 0.06 m/s) positive bias that may be due to a systematic error related to the characteristics of the acquisition protocol (e.g., time estimation with a stopwatch).

The obtained results demonstrate the feasibility and validity of the presented device for the measurement of real-world walking speed, even in tracts with high interference. The main advantage of such device, with respect to other devices currently used for real-world walking speed estimation such as IMUs (Mazzà et al., 2021), is the fact that a wide set of additional measures and metrics (not measurable by an IMU) can be obtained. GPS devices in fact can provide, thanks to their localization capabilities, quantitative information on several aspects characterizing daily mobility such as life space (Fillekes et al., 2019; Taylor et al., 2019), out-of-home activities (Fillekes et al., 2019; Haeger et al., 2022), active transport modes (Fillekes et al., 2019), trajectories (Ziepert et al., 2021), distances from specific points (or participants) (Ziepert et al., 2021), time spent indoor/outdoor (Bayat et al., 2022), and type of activities (e.g. medical or sport-related) (Bayat et al., 2022).

The first limitation of this study is that we only tested a single weather condition (sunny and cloudless). This allowed us to focus on the effect of landscape interference, but further studies are needed considering interference from different weather situations (e.g., cloudy vs. sunny), which can also affect the system’s accuracy.

Another limitation of this study is the small sample size which was used for this exploratory study. Larger sample sizes should be considered in future studies. A further limitation is that only healthy adults were considered, although this is common to most studies using GPS devices for walking speed estimation, except from two exploratory studies where a non-differential GPS was used to evaluate walking speed in people with Multiple Sclerosis (Delahaye et al., 2021) and people with claudication (Gernigon et al., 2014).

As a further future development, the reference system could be improved to be more independent from human error than a stopwatch, although this kind of system is often used in similar works.

Also, a usability analysis of the presented system on clinical populations of interest is an important future step to perform. In fact, this device is not as easily wearable as a smartwatch or an IMU (although being much smaller than RTK survey-grade devices).

Another possible future development is using a sensor fusion approach to integrate the functionality of the RTK-GPS receiver with an inertial sensor or other kinds of devices (Barry et al., 2018). This union could overcome the shortcomings of the GPS (the need to be used outdoors and the degradation of accuracy in situations with high interference). On the other hand, the information provided by the GPS, like true position and absolute time reference, could be beneficial for increasing an IMU functionality.

In conclusion, this is the first study that uses a mass-market RTK receiver to measure and validate WS in a real-world scenario to the best of our knowledge. The obtained results provide a preliminary insight and validation of the potential of a mass-market RTK receiver to measure walking speed in the real world. Notably, this work has proven the suitability of the simpleRTK2B for measuring average real-world walking speed even in environments with high interference and poor sky visibility. The measurements obtained in healthy adults were accurate enough to measure clinically important differences.

## Data Availability

The raw data supporting the conclusions of this article will be made available by the authors, without undue reservation.

## References

[B1] Abellan Van KanG.RollandY.AndrieuS.BauerJ.BeauchetO.BonnefoyM. (2009). Gait Speed at Usual Pace as a Predictor of Adverse Outcomes in Community-Dwelling Older People an International Academy on Nutrition and Aging (IANA) Task Force. J. Nutr. Health Aging 13 (10), 881–889. 10.1007/S12603-009-0246-Z 19924348

[B2] Barry L. C. Hatchman L. Fan Z. Guralnik J. M. Gao R. X. Kuchel G. A. (2018). Design and Validation of a Radio‐Frequency Identification‐Based Device for Routinely Assessing Gait Speed in a Geriatrics Clinic. J. Am. Geriatr. Soc. 66, 982–986. 10.1111/JGS.15315 29473949

[B3] Bayat S. Naglie G. Rapoport M. J. Stasiulis E. Widener M. J. Mihailidis A. (2022). A GPS-Based Framework for Understanding Outdoor Mobility Patterns of Older Adults with Dementia: An Exploratory Study. Gerontology 68, 1–15. 10.1159/000515391 33895746

[B4] Bland J. M. Altman D. G. (1999). Measuring Agreement in Method Comparison Studies. Stat. Methods Med. Res. 8, 135–160. 10.1177/09622802990080020410.1191/096228099673819272 10501650

[B5] Bohannon R. W. Glenney S. S. (2014). Minimal Clinically Important Difference for Change in Comfortable Gait Speed of Adults with Pathology: a Systematic Review. J. Eval. Clin. Pract. 20, 295–300. 10.1111/jep.12158 24798823

[B6] Dammalage T. L. Samarakoon L. (2008). Test Results of Rtk and Real-Time Dgps Corrected Observations Based on Ntrip Protocol. Int. Arch. Photogramm Remote Sens Spat. Inf. Sci. XXXVII, 1119–1124. Available at: http://citeseerx.ist.psu.edu/viewdoc/download?doi=10.1.1.155.4801&rep=rep1&type=pdf (Accessed January 14, 2022).

[B7] Delahaye C. Chaves D. Congnard F. Noury-Desvaux B. de Müllenheim P.-Y. (2021). Measuring Outdoor Walking Capacities Using Global Positioning System in People with Multiple Sclerosis: Clinical and Methodological Insights from an Exploratory Study. Sensors 21, 3189. 10.3390/s21093189 34064381 PMC8125650

[B8] Ferrucci L. Bandinelli S. Benvenuti E. Di Iorio A. Macchi C. Harris T. B. (2000). Subsystems Contributing to the Decline in Ability to Walk: Bridging the Gap between Epidemiology and Geriatric Practice in the InCHIANTI Study. J. Am. Geriatr. Soc. 48, 1618–1625. 10.1111/J.1532-5415.2000.TB03873.X 11129752

[B9] Fillekes M. P. Giannouli E. Kim E.-K. Zijlstra W. Weibel R. (2019). Towards a Comprehensive Set of GPS-Based Indicators Reflecting the Multidimensional Nature of Daily Mobility for Applications in Health and Aging Research. Int. J. Health Geogr. 18, 17. 10.1186/s12942-019-0181-0 31340812 PMC6657041

[B10] Fritz S. Lusardi M. (2009). White Paper: "Walking Speed: the Sixth Vital Sign". J. Geriatr. Phys. Ther. 32, 2–5. 10.1519/00139143-200932020-00002 20039582

[B11] Gernigon M. Fouasson-Chailloux A. Colas-Ribas C. Noury-Desvaux B. Le Faucheur A. Abraham P. (2015). Test-retest Reliability of GPS Derived Measurements in Patients with Claudication. Eur. J. Vasc. Endovascular Surg. 50, 623–629. 10.1016/j.ejvs.2015.07.009 26319478

[B12] Gernigon M. Le Faucheur A. Noury-Desvaux B. Mahe G. Abraham P. (2014). Applicability of Global Positioning System for the Assessment of Walking Ability in Patients with Arterial Claudication. J. Vasc. Surg. 60, 973–981. e1. 10.1016/J.JVS.2014.04.053 24930016

[B13] Goldie P. A. Matyas T. A. Evans O. M. (1996). Deficit and Change in Gait Velocity during Rehabilitation after Stroke. Arch. Phys. Med. Rehabil. 77, 1074–1082. 10.1016/S0003-9993(96)90072-6 8857890

[B14] Graham J. E. Ostir G. V. Fisher S. R. Ottenbacher K. J. (2008). Assessing Walking Speed in Clinical Research: a Systematic Review. J. Eval. Clin. Pract. 14, 552–562. 10.1111/J.1365-2753.2007.00917.X 18462283 PMC2628962

[B15] Guimaraes R. M. Isaacs B. (1980). Characteristics of the Gait in Old People Who Fall. Int. Rehabil. Med. 2 (4), 177–180. 10.3109/09638288009163984 7239777

[B16] Haeger C. Mümken S. A. O‘Sullivan J. L. Spang R. P. Voigt-Antons J.-N. Stockburger M. (2022). Mobility Enhancement Among Older Adults 75 + in Rural Areas: Study Protocol of the MOBILE Randomized Controlled Trial. BMC Geriatr. 22, 22. 10.1186/S12877-021-02739-0 35057755 PMC8771178

[B17] Haghayegh S. Kang H.-A. Khoshnevis S. Smolensky M. H. Diller K. R. Diller K. R. (2020). A Comprehensive Guideline for Bland-Altman and Intra Class Correlation Calculations to Properly Compare Two Methods of Measurement and Interpret Findings. Physiol. Meas. 41, 055012. 10.1088/1361-6579/ab86d6 32252039

[B18] Hardy S. E. Perera S. Roumani Y. F. Chandler J. M. Studenski S. A. (2007). Improvement in Usual Gait Speed Predicts Better Survival in Older Adults. J. Am. Geriatr. Soc. 55, 1727–1734. 10.1111/j.1532-5415.2007.01413.x 17916121

[B19] Kaplan E. Hegarty C. (2005). Understanding GPS Principles and Applications : Principles and Applications. Norwood, MA: Artech House, 723.

[B20] King L. A. Salarian A. Mancini M. Priest K. C. Nutt J. Serdar A. (2013). Exploring Outcome Measures for Exercise Intervention in People with Parkinson's Disease. Parkinson's Dis. 2013, 1–9. 10.1155/2013/572134 PMC3657411 23738230

[B21] Klous G. Smit L. A. M. Borlée F. Coutinho R. A. Kretzschmar M. E. E. Heederik D. J. J. (2017). Mobility Assessment of a Rural Population in the Netherlands Using GPS Measurements. Int. J. Health Geogr. 16, 30–13. 10.1186/S12942-017-0103-Y/FIGURES/4 28793901 PMC5551017

[B22] Le Faucheur A. Abraham P. Jaquinandi V. Bouyé P. Saumet J. L. Noury-Desvaux B. (2007). Study of Human Outdoor Walking with a Low-Cost GPS and Simple Spreadsheet Analysis. Med. Sci. Sports Exerc. 39, 1570–1578. 10.1249/mss.0b013e3180cc20c7 17805090

[B23] Mazzà C. Alcock L. Aminian K. Becker C. Bertuletti S. Bonci T. (2021). Technical Validation of Real-World Monitoring of Gait: a Multicentric Observational Study. BMJ Open 11, e050785. 10.1136/BMJOPEN-2021-050785 PMC8640671 34857567

[B24] Montero-Odasso M. Schapira M. Soriano E. R. Varela M. Kaplan R. Camera L. A. (2005). Gait Velocity as a Single Predictor of Adverse Events in Healthy Seniors Aged 75 Years and Older. J. Gerontol. A. Biol. Sci. Med. Sci. 60 (10), 1304–1309. 10.1093/gerona/60.10.1304 16282564

[B25] Moseley A. M. Lanzarone S. Bosman J. M. Van Loo M. A. De Bie R. A. Hassett L. (2004). Ecological Validity of Walking Speed Assessment after Traumatic Brain Injury. J. Head Trauma Rehabil. 19, 341–348. 10.1097/00001199-200407000-00008 15263861

[B26] Newman A. B. Simonsick E. M. Naydeck B. L. Boudreau R. M. Kritchevsky S. B. Nevitt M. C. (2006). Association of Long-Distance Corridor Walk Performance with Mortality, Cardiovascular Disease, Mobility Limitation, and Disability. JAMA 295, 2018–2026. 10.1001/JAMA.295.17.2018 16670410

[B27] Noury-Desvaux B. Abraham P. Mahé G. Sauvaget T. Leftheriotis G. Le Faucheur A. (2011). The Accuracy of a Simple, Low-Cost GPS Data Logger/Receiver to Study Outdoor Human Walking in View of Health and Clinical Studies. PLoS One 6, e23027. 10.1371/JOURNAL.PONE.0023027 21931593 PMC3172201

[B28] Perry J. Garrett M. Gronley J. K. Mulroy S. J. (1995). Classification of Walking Handicap in the Stroke Population. Stroke 26, 982–989. 10.1161/01.STR.26.6.982 7762050

[B29] Purser J. L. Weinberger M. Cohen H. J. Pieper C. F. Morey M. C. Li T. (2005). Walking Speed Predicts Health Status and Hospital Costs for Frail Elderly Male Veterans. J. Rehabil. Res. Dev. 42, 535–546. 10.1682/JRRD.2004.07.0087 16320148

[B30] Schimpl M. Moore C. Lederer C. Neuhaus A. Sambrook J. Danesh J. (2011). Association between Walking Speed and Age in Healthy, Free-Living Individuals Using Mobile Accelerometry-A Cross-Sectional Study. PLoS One 6, e23299. 10.1371/journal.pone.0023299 21853107 PMC3154324

[B31] Stellmann J. P. Neuhaus A. Götze N. Briken S. Lederer C. Schimpl M. (2015). Ecological Validity of Walking Capacity Tests in Multiple Sclerosis. PLoS One 10, e0123822. 10.1371/JOURNAL.PONE.0123822 25879750 PMC4399985

[B32] StudenskiS. (2009). Bradypedia: Is Gait Speed Ready for Clinical Use. J. Nutr. Health Aging 13, 878–880. 10.1007/S12603-009-0245-0 19924347

[B33] Studenski S. (2011). Gait Speed and Survival in Older Adults. JAMA 305, 50. 10.1001/jama.2010.1923 21205966 PMC3080184

[B34] Studenski S. Perera S. Wallace D. Chandler J. M. Duncan P. W. Rooney E. (2003). Physical Performance Measures in the Clinical Setting. J. Am. Geriatr. Soc. 51, 314–322. 10.1046/J.1532-5415.2003.51104.X 12588574

[B35] Taylor J. K. Buchan I. E. van der Veer S. N. (2019). Assessing Life-Space Mobility for a More Holistic View on Wellbeing in Geriatric Research and Clinical Practice. Aging Clin. Exp. Res. 31, 439–445. 10.1007/S40520-018-0999-5/TABLES/2 30078096 PMC6439151

[B36] Teunissen P. J. G. (2003). Theory of Carrier Phase Ambiguity Resolution. Wuhan Univ. J. Nat. Sci. 8, 471–484. 10.1007/BF02899809

[B37] Townshend A. D. Worringham C. J. Stewart I. B. (2008). Assessment of Speed and Position during Human Locomotion Using Nondifferential GPS. Med. Sci. Sports Exerc. 40, 124–132. 10.1249/MSS.0B013E3181590BC2 18091013

[B38] u-blox F9 Hpg (2021). U-Blox F9 High Precision GNSS Receiver Interface Description. Available at: https://www.u-blox.com/sites/default/files/u-blox-F9-HPG-1.30_InterfaceDescription_UBX-21046737.pdf (Accessed January 14, 2022).

[B39] Yildiz M. (2012). The Impact of Slower Walking Speed on Activities of Daily Living in Patients with Multiple Sclerosis. Int. J. Clin. Pract. 66, 1088–1094. 10.1111/IJCP.12003 23067032 PMC3506731

[B40] Ziepert B. de Vries P. W. Ufkes E. (2021). Psyosphere”: A GPS Data-Analysing Tool for the Behavioural Sciences. Front. Psychol. 12, 1650. 10.3389/FPSYG.2021.538529/BIBTEX PMC8155254 34054626

